# Glenohumeral Instability and Clinical Outcomes Following Proximal Humerus Resection and Megaprosthesis Implantation: A Systematic Review

**DOI:** 10.3390/jcm14217850

**Published:** 2025-11-05

**Authors:** Luigi Cianni, Giacomo Capece, Luca Fiore, Andrea De Fazio, Sara Martellini, Giulio Maccauro, Maristella Francesca Saccomanno

**Affiliations:** 1 Orthopaedics and Trauma Surgery Unit, Catholic University of the Sacred Heart, 00168 Rome, Italy; luigi.cianni1@guest.policlinicogemelli.it (L.C.); fioreluca1999@gmail.com (L.F.); andrea.defazio01@icatt.it (A.D.F.); sara.martellini01@icatt.it (S.M.); giulio.maccauro@unicatt.it (G.M.); 2 Orthopaedics and Trauma Surgery Unit, Department of Ageing, Neurosciences, Head-Neck and Orthopaedics Sciences, Fondazione Policlinico Universitario Agostino Gemelli IRCCS, 00168 Rome, Italy; maristellasaccomanno@hotmail.it; 3 Orthopaedics and Trauma Surgery Unit, Pellegrini Hospital, 80134 Naples, Italy

**Keywords:** proximal humerus, megaprosthesis, glenohumeral instability, shoulder surgery, oncologic resection, osteosarcoma, chondrosarcoma, bone metastasis

## Abstract

**Background:** Glenohumeral instability is one of the most frequent and clinically impactful complications following proximal humerus resection and reconstruction with a megaprosthesis, especially in patients treated for bone tumors or complex fractures. Its incidence, risk factors, and influence on functional recovery remain variably reported in the literature. **Methods**: A systematic review was conducted according to PRISMA guidelines, searching PubMed, Scopus, and Google Scholar up to April 2025. Studies reporting on postoperative instability, dislocation, functional outcomes (MSTS, DASH), and related complications were included. Two independent reviewers performed data extraction and quality assessment. A pooled analysis was performed using random-effects models. **Results:** A total of 17 studies including 387 patients were analyzed. The pooled incidence of glenohumeral instability was 32%, with a revision surgery rate of 10% due to instability. The most common reconstruction technique was modular megaprosthesis (47%), followed by allograft–prosthesis composites (APCs) and reverse total shoulder arthroplasty (RSA). Functional outcomes were reported in 12 studies using the Musculoskeletal Tumor Society (MSTS) score, with a weighted mean of 22.3 ± 3.8 (74.3% ± 12.7%). Disabilities of the Arm, Shoulder, and Hand (DASH) scores, reported in 3 studies, showed worse outcomes in unstable shoulders (mean 61.4 ± 5.2 vs. 26.6 ± 4.1). Soft tissue reconstruction, particularly involving the rotator cuff and deltoid, significantly influenced postoperative stability and function. **Conclusions:** Glenohumeral instability after proximal humerus megaprosthesis is a common and disabling complication that adversely affects functional outcomes and revision rates. Optimizing soft tissue management and prosthetic design is essential to improve joint stability and long-term results.

## 1. Introduction

The surgical management of primary malignant tumors and unreconstructable metastatic lesions of the proximal humerus often necessitates resection of the proximal humerus followed by reconstruction with a megaprosthesis, a fundamental approach in limb salvage procedures for both primary malignant bone tumors (such as osteosarcoma, chondrosarcoma, and Ewing’s sarcoma) and metastatic lesions involving the proximal humerus [1]. In addition, patients presenting with complex fractures characterized by metaphyseal extension and significant bone loss, as well as those requiring revision shoulder arthroplasty with bone loss or treatment of periprosthetic fractures, may also benefit from megaprosthetic reconstruction to restore structural integrity and preserve upper limb function [2]. Despite considerable advances in surgical techniques, prosthetic design, and perioperative management, postoperative complications remain a significant concern. Among these, glenohumeral instability represents one of the most frequent and clinically challenging complications following proximal humerus resection and megaprosthesis implantation [3]. This instability can lead to pain, limited range of motion, decreased shoulder function, and ultimately diminished quality of life for patients [4,5].

A wide array of prosthetic options are currently utilized in clinical practice, reflecting the heterogeneity of tumor pathology and patient-specific anatomical considerations. These include modular tumor prostheses, allograft–prosthesis composites (APCs), customized implants, and reverse total shoulder arthroplasties (RSA) [6,7]. Each implant type presents unique advantages and challenges with regard to fixation, soft tissue attachment, and functional outcomes [8]. Soft tissue management strategies play a critical role in stabilizing the prosthesis and restoring shoulder mechanics [9]. Techniques range from meticulous reattachment of remaining musculature and tendons to the use of synthetic materials such as Trevira tubes or Dacron tapes to augment soft tissue support and prosthesis suspension [10,11,12].

Despite a growing body of literature, there remains significant heterogeneity in the reported incidence of glenohumeral instability, the identification of risk factors, and the impact on functional recovery following proximal humerus megaprosthesis implantation. Furthermore, a consensus on best practices for surgical technique, soft tissue management, and rehabilitation to minimize instability has not been established.

Given these challenges, a systematic review of the available evidence is warranted to better characterize the incidence and clinical impact of glenohumeral instability in this context. Such an analysis can also help identify modifiable risk factors and inform surgical decision-making. The primary aim of this study is to provide a comprehensive synthesis of clinical outcomes related to instability after proximal humerus resection and megaprosthesis implantation, evaluating both the functional consequences and the efficacy of various surgical interventions aimed at improving joint stability. Ultimately, these insights will contribute to optimizing patient care and improving long-term outcomes for this complex and vulnerable patient population.

## 2. Materials and Methods

Standard systematic review methods were employed in this study. The review process was conducted in accordance with the PRISMA (Preferred Reporting Items for Systematic Reviews and Meta-Analyses) [13] guidelines to ensure a rigorous and transparent selection and evaluation of the literature (See Supplementary Materials for the checklist). The protocol was registered with the International Prospective Register of Systematic Reviews (PROSPERO) under the registration number 1048373. The review process was conducted up to April 2025.

The literature search was carried out independently by two authors (GC and LF) across three major electronic databases: PubMed, Google Scholar, Web of sciences and Scopus. Additionally, we manually screened references of included studies to identify potentially relevant grey literature, conference abstracts, and unpublished data.

To identify all potentially relevant studies, we used a comprehensive search strategy that included both MeSH terms and free-text keywords. The search covered all studies published from the inception of each database until April 2025. Google Scholar was also searched to capture additional peer-reviewed studies and relevant grey literature. The following search string was applied: ((shoulder joint[All Fields] OR glenohumeral joint[All Fields] OR Shoulder Joint[MeSH]) AND (proximal humeral resection[All Fields] OR proximal humerus resection[All Fields] OR proximal humerus surgery[All Fields] OR tumor resection[All Fields] OR Humerus[MeSH] OR Bone Neoplasms/surgery[MeSH]) AND (megaprosthesis[All Fields] OR shoulder prosthesis[All Fields] OR joint replacement[All Fields] OR Joint Prosthesis[MeSH]) AND (shoulder instability[All Fields] OR glenohumeral instability[All Fields] OR shoulder dislocation[All Fields] OR instability[All Fields] OR Shoulder Dislocation[MeSH] OR Joint Instability[MeSH]) AND (rate[All Fields] OR recurrence[All Fields] OR outcome[All Fields] OR complications[All Fields])).

**Figure 1:**
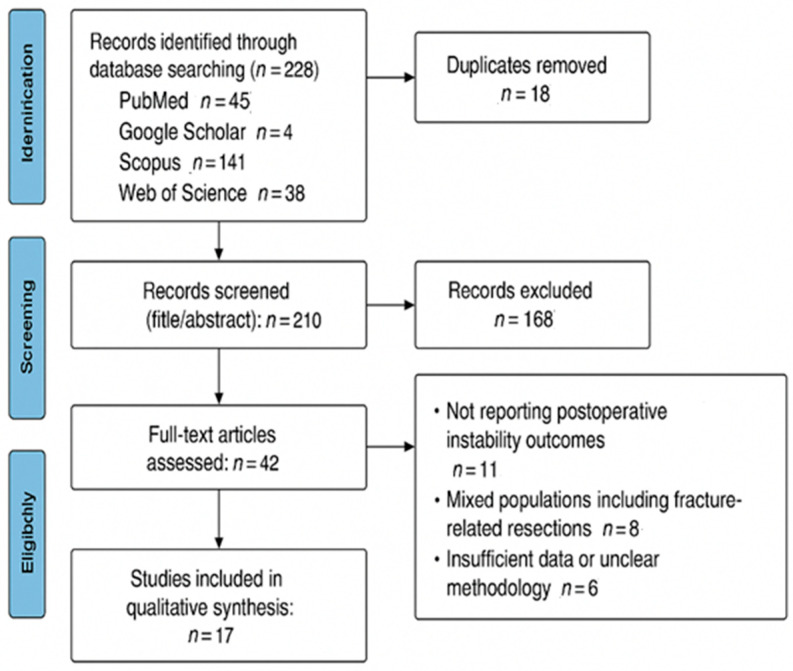
PRISMA flow-chart. Flow diagram illustrating the study selection process, including the number of records identified, screened, excluded, and included in the final analysis.

The literature search was conducted across four electronic databases: PubMed (n = 45), Google Scholar (n = 42), Scopus (n = 141), and Web of Science (n = 0, no additional eligible results). In addition, reference lists of the included articles were manually screened to identify potentially relevant grey literature and conference abstracts. The study selection process is summarized in the PRISMA flow diagram (Figure 1).

Eligibility criteria were clearly defined to ensure the inclusion of studies relevant to glenohumeral instability and clinical outcomes following proximal humerus resection and megaprosthesis implantation. We included both retrospective and prospective clinical studies, including randomized controlled trials, which reported on patients who underwent proximal humeral resection with megaprosthesis reconstruction. After further review, only studies addressing tumor-related resections were included. We considered only studies published in English to ensure consistent data interpretation. To guarantee a sufficient sample size for meaningful data analysis, studies with fewer than five patients were excluded. Case reports, reviews, meta-analyses, in vitro or cadaveric studies, and studies reporting conditions unrelated to glenohumeral instability were also excluded. Data extraction was performed independently by two authors (GC and LF) using a predefined Excel sheet (Microsoft Corporation. Microsoft Excel. Version 16.0. Redmond, WA, USA: Microsoft; 2021). Extracted variables included histological tumor type, duration of follow-up (in months), type of treatment performed (e.g., endoprosthesis, reverse prosthesis), soft tissue management strategy, resection length (in centimeters), rehabilitation protocols, and outcome scores such as MSTS and DASH. Postoperative glenohumeral instability was defined as any clinical or radiographic evidence of shoulder subluxation or dislocation, including both symptomatic cases requiring intervention and cases reported as “instability” in the source study. We recorded whether instability was present (yes/no), the number of instability events, any associated complications, whether surgical revision for instability was required, and the number and type of such revisions (Table 1). This approach allowed us to include all relevant instability events, regardless of whether they were treated surgically or conservatively. Disagreements between the two reviewers during data extraction were resolved by discussion, and if consensus could not be reached, a senior author (LC) was consulted to make the final decision.

To assess the methodological quality and risk of bias of the included studies, we employed the Methodological Index for Non-Randomized Studies (MINORS) tool [14,15]. Since all included studies were non-randomized retrospective case series (Level IV evidence), the MINORS score was used as the sole instrument for quality assessment, and the Cochrane Risk of Bias tool was not applied. The MINORS tool consists of 12 items, each scored from 0 to 2, with a maximum of 16 points for non-comparative studies and 24 points for comparative studies. Two reviewers (GC and LF) independently assessed each study, and any disagreements were resolved by consensus with a senior author (LC). The assessed domains included clarity of aims, inclusion of consecutive patients, prospective data collection, appropriate endpoints, unbiased outcome assessment, adequate follow-up, acceptable loss to follow-up, and appropriate statistical analyses. The total MINORS score for each study was calculated and summarized in a dedicated table (Table 2).

All the 17 included studies were retrospective (100%) and classified as Level IV evidence.

**Table 1:** Data extraction.

Authors	Study Design	Level of Evidence	Number of Patients	Follow-Up (Months)	Mean Age	Gender (M/F)	Primary Histology	MINORS Score/Cochrane Risk of Bias (RoB)	Post Operative Instability	Number of Cases of Postoperative Instability
El Beaino et al. [16]	Retrospective	IV	21	97	41	14/7	Chondrosarcoma, Osteosarcoma	14	Yes	12
Hartigan et al. [17]	Retrospective	IV	30	76.8	43.8	14/13	n/s	13	Yes	7
Rahman et al. [18]	Retrospective	IV	10	61	36	n/s	Chondrosarcoma, Osteosarcoma	14	No	0
Wang et al. [19]	Retrospective	IV	18	56	29.8	8/10	Osteosarcoma, Chondrosarcoma	13	Yes	1
Van de Sande et al. [20]	Retrospective	IV	37	120	44.8	21/16	Osteosarcoma, Chondrosarcoma	14	Yes	2
Wang et al. [21]	Retrospective	IV	16	27.4	45.9	7/9	Metastatic, Chondrosarcoma	12	No	0
Black et al. [22]	Retrospective	IV	6	25.2	40.7	2/4	Chondrosarcoma	14	Yes	5
Wang et al. [23]	Retrospective	IV	25	48	32	10/15	Chondrosarcoma, Osteosarcoma	15	Yes	10
El Motassime et al. [24]	Retrospective	IV	20	21	61.3	12/8	Metastatic tumor	20	Yes	2
Rachbauer AM et al. [25]	Retrospective	IV	46	25	52	15/31	Chondrosarcoma, Osteosarcoma, Ewing’s sarcoma, Giant Cell Tumor, Metastatic	14	Yes	8
Errani et al. [26]	Retrospective	IV	18	56.4	n/s	9/9	n/s	14	Yes	4
Vonck et al. [27]	Retrospective	IV	20	18.3	55.3	8/12	Liposarcoma, Osteosarcoma, Metastatic	13	Yes	4
Shi et al. [28]	Retrospective	IV	18	29	37	9/9	Osteosarcoma, Chondrosarcoma	13	Yes	2
Fucentese et al. [29]	Retrospective	IV	30	24	63.3	19/11	n/s	13	Yes	2
Tagliero et al. [30]	Retrospective	IV	33	96	67	17/16	Osteosarcoma, Soft tissue sarcoma	14	Yes	5
Hu et al. [31]	Retrospective	IV	7	23.6	34.9	3/4	Osteosarcoma, Chondrosarcoma	12	No	0
Raiss et al. [32]	Retrospective	IV	39	38	60	19/20	Metastatic + Primary	15	yes	4

**Table 2:** Summary of methodological quality of included studies.

Study	Study Design	Level of Evidence	MINORS Total Score (0–16)	Quality Category
El Beaino et al. [16]	Retrospective	IV	14	Moderate
Hartigan et al. [17]	Retrospective	IV	13	Moderate
Rahman et al. [18]	Retrospective	IV	14	Moderate
Wang et al. [19]	Retrospective	IV	13	Moderate
Van de Sande et al. [20]	Retrospective	IV	14	Moderate
Wang et al. [21]	Retrospective	IV	12	Moderate–Low
Black et al. [22]	Retrospective	IV	14	Moderate
Wang et al. [23]	Retrospective	IV	15	Moderate–High
El Motassime et al. [24]	Retrospective	IV	20	High (comparative study)
Rachbauer et al. [25]	Retrospective	IV	14	Moderate
Errani et al. [26]	Retrospective	IV	14	Moderate
Vonck et al. [27]	Retrospective	IV	13	Moderate
Shi et al. [28]	Retrospective	IV	13	Moderate
Fucentese et al. [29]	Retrospective	IV	13	Moderate
Tagliero et al. [30]	Retrospective	IV	14	Moderate
Hu et al. [31]	Retrospective	IV	12	Moderate–Low
Raiss et al. [32]	Retrospective	IV	15	Moderate–High

## 3. Results

A total of 17 studies were included in the pooled analysis, comprising 16 retrospective and 1 prospective study. The majority of studies were classified as level IV evidence (n = 15), with one study of level III evidence. Overall, these studies included 387 patients who underwent reconstructive shoulder surgery following tumor resection.

The mean age was reported in 14 studies and was 43.7 years (SD ± 13.2), with an age range from 8 to 90 years. Gender distribution was available in 16 studies, comprising 189 males (48.8%) and 198 females (51.2%). Follow-up data were reported in 17 studies, with a mean duration of 54.3 months (SD ± 36.4) and a range from 13 to 120 months.

Histological classification of the primary tumors was reported in 14 studies, showing a predominance of osteosarcoma (25.3%) and chondrosarcoma (22.0%), followed by giant cell tumor (12.1%) and metastatic lesions (15.0%). The remaining 25.6% included a variety of less common neoplasms, such as Ewing’s sarcoma, multiple myeloma, lymphomas, desmoplastic fibroma, malignant fibrous histiocytoma, and other soft tissue sarcomas (Table 1).

Regarding the surgical approach, 8 studies investigated modular tumor prostheses, 4 studies investigated allograft–prosthesis composites, 1 study investigated reverse total shoulder arthroplasty, 2 studies investigated both modular tumor prostheses and allograft–prosthesis composites, and 1 study reported on prosthetic replacement not otherwise specified.

The most frequently employed method was modular tumor prosthesis implantation, utilized in 47% of cases. Composite reconstructions using an allograft0150prosthesis technique accounted for 29%, while RSA was used in 14% of patients. Custom-made prosthetic designs were adopted in 10% of cases.

The mean bone resection length, reported in 10 studies, was 12.3 cm (SD ± 3.8), with the shortest resections being 4 cm and the longest extending up to 35 cm.

Management of soft tissues varied considerably among the surgical strategies and was reported in 14 studies. Rotator cuff and deltoid repair were performed in 5 studies (approximately 65% of patients), often involving reattachment to either the allograft or surrounding fascia. In more complex resections, particularly in procedures where the deltoid or rotator cuff were compromised or sacrificed, advanced reconstruction techniques such as Trevira tube anchoring and the use of synthetic meshes or Dacron tapes were adopted. This technique was cited in 5 studies (35% of cases).

Postoperative rehabilitation protocols was reported in 14 studies. It typically consisted of an initial immobilization phase lasting between 4 and 7 weeks, followed by structured physiotherapy to gradually restore function.

Each study included in the review analyzed complications. The most common complications encountered postoperatively included instability, which was observed in 122 patients, representing 32% of the overall population. When weighted by sample size, the pooled incidence of instability was 31.5% (95% CI, 27.0–36.2%). Revision surgery due to instability was required in 39 patients, accounting for 10% of the total cohort. Other complications were also frequently reported, including infections (7%), aseptic loosening of the implant, periprosthetic fractures, radial or brachial plexus nerve injuries, and local tumor recurrence. Subgroup analysis by implant type was performed. Instability rates were highest in patients treated with modular tumor prostheses (34%, 95% CI 28.0–40.5%), followed by APC (28%, 95% CI 22.4–34.9%), and lowest in RSA (18%, 95% CI 10.6–28.5%). When stratified by tumor type, instability was higher in osteosarcoma (36%) and chondrosarcoma (31%) than in metastatic lesions (22%).

The overall revision surgery rate across all patients was 22%, with higher frequencies observed in cases involving modular prostheses or allograft–prosthetic composite constructs. In these patients, the need for secondary procedures was often attributed to mechanical failures, such as prosthetic loosening or instability, as well as biological issues including non-union, infections, or tumor relapses. Several studies reported the use of conversion to RSA as a salvage procedure in cases of failed soft tissue repairs or recurrent dislocation.

Functional outcomes were evaluated using various scoring systems across the included studies. The Musculoskeletal Tumor Society (MSTS) score was the most commonly reported, used in 12 studies. The overall weighted mean MSTS score across these 12 studies was 22.3 ± 3.8 (74.3% ± 12.7%), based on a combined total of 312 patients. The MSTS scores ranged from 12/30 (40%) to 27/30 (90%). Some studies reported excellent results in well-functioning cases (up to 27/30, 90%), while others documented more modest outcomes, particularly in older patients or in those undergoing extensive soft tissue resections. The Disabilities of the Arm, Shoulder, and Hand (DASH) score was reported in 3 studies, with a pooled mean of 61.4 ± 5.2 in unstable shoulders and 26.6 ± 4.1 in stable shoulders, indicating a variable impact on upper limb function depending on patient and surgical factors. Scores reported in fewer than two studies, such as the Constant score, ASES, Enneking, and International Society of Limb Salvage scores, were not included in the pooled analysis (Table 3). However, the comparison of functional outcomes between stable and unstable shoulders is based on limited data derived from only two studies.

## 4. Discussion

This review provides a comprehensive overview of reconstructive strategies following shoulder tumor resection across a diverse patient population. With 387 patients and a mean follow-up exceeding four years, the data offer valuable insight into the oncologic and functional outcomes of various reconstructive modalities. The cohort was heterogeneous with respect to age, tumor histology, extent of resection, and surgical approach, reflecting the complexity and variability inherent in musculoskeletal oncology.

The predominance of osteosarcoma and chondrosarcoma aligns with existing epidemiological patterns of primary malignant bone tumors affecting the shoulder girdle [33]. The inclusion of metastatic and rare tumor types underscores the need for adaptable and personalized surgical strategies capable of addressing both curative and palliative goals.

From a surgical standpoint, modular tumor prostheses were the most frequently employed technique. Their popularity likely stems from their intraoperative versatility and relative ease of use, particularly in the setting of variable resection lengths [34]. However, the analysis highlights a non-negligible incidence of mechanical complications, particularly instability, which affected nearly one-third of the overall cohort. This complication was not only frequent but also clinically significant, as reflected by the substantial difference in DASH scores between stable and unstable reconstructions. It is important to recognize that instability is a spectrum and is strongly influenced by the residual soft tissue and muscular function. Many patients included in this review had partial or absent deltoid and/or rotator cuff function due to tumor resection, or nerve injuries affecting deltoid activation. Consequently, some degree of instability in these patients is expected and should be interpreted in the context of their anatomical and functional limitations.

Approximately 35% of patients underwent advanced soft tissue anchoring using synthetic devices or tendon reconstructions. Among patients with adequate soft tissue reconstruction, instability was infrequent, whereas the majority of postoperative dislocations occurred in cases with partial or absent soft tissue repair. Overall, 9 of 36 patients (25%) experienced postoperative instability, highlighting the protective role of soft tissue reconstruction in preventing dislocations and reducing the need for revision surgery. These findings suggest that inclusion of patients with non-functioning deltoids or nerve palsies likely contributes to the observed incidence of instability, and such cases represent a distinct subgroup with higher mechanical vulnerability. From a biomechanical perspective, shoulder instability following tumor resection and reconstruction is multifactorial. Insufficient deltoid tensioning can lead to loss of compressive force across the glenohumeral articulation, predisposing to dislocation, particularly in cases where the deltoid origin or insertion has been resected or inadequately restored [24].

In addition, alterations in humeral offset or center of rotation, common after modular or allograft–prosthesis reconstructions, can change the lever arm of the deltoid and rotator cuff muscles, reducing joint stability and functional strength. Restoring an appropriate humeral offset and maintaining the physiological center of rotation are therefore critical to optimize soft tissue balance and minimize the risk of instability. Furthermore, the use of reverse shoulder designs, which medialize and distalize the center of rotation, may compensate for deficient rotator cuff function by enhancing deltoid efficiency, explaining the lower dislocation rates observed in these reconstructions [35].

Composite reconstructions with allograft–prosthesis combinations provided an alternative approach, particularly in younger patients or when extensive bony resection was required. Although theoretically advantageous for biological integration and muscle reattachment, these constructs were also associated with relatively high revision rates, often due to non-union, fracture, or infection [36]. RSA, while less frequently used as a primary reconstruction, emerged as a valuable salvage option, especially in cases of failed soft tissue reconstructions or recurrent dislocation. RSA relies primarily on deltoid function rather than an intact rotator cuff, making it a biologically suitable alternative in selected patients, particularly older individuals or those with limited functional demands [37].

Functional outcomes, as measured by the MSTS and DASH scores, demonstrated a wide spectrum. While the average MSTS score was 74.3%, reflecting moderate to good function in most patients, the variability among studies highlights the impact of surgical technique, patient selection, and complication profile. Notably, the poorest outcomes were associated with instability, confirming it as a major determinant of patient-reported disability and overall satisfaction.

The overall revision rate of 22% is comparable to or slightly higher than previously reported rates in limb-salvage surgery literature, likely reflecting the complexity of the shoulder joint and the biomechanical demands placed upon it [38]. In particular, modular and composite prosthetic reconstructions appeared more prone to mechanical failure, especially when extensive soft tissue loss was present.

This analysis is not without limitations. The included studies exhibited methodological heterogeneity, particularly regarding reporting standards, definitions of complications, and functional outcome measures. Most studies were retrospective and lacked comparisons between reconstruction types, limiting the ability to draw definitive conclusions about the superiority of one technique over another. Additionally, the diversity in tumor types, patient age, and extent of resection further complicates comparisons. Moreover, the comparison of functional outcomes between stable and unstable shoulders is based on data derived from only two studies, which limits the strength of this finding and warrants cautious interpretation.

## 5. Conclusions

Postoperative instability remains the major challenge in proximal humerus reconstruction following tumor resection. This pooled analysis highlights that, although modular and composite prostheses provide acceptable oncologic control and functional outcomes, they are frequently complicated by instability, representing the primary determinant of functional failure and the leading cause of revision surgery.

Effective management of soft tissues, particularly the deltoid and rotator cuff, is crucial to minimizing instability and improving postoperative shoulder function. Despite recent advances in surgical techniques and implant design, the incidence of instability remains considerable, underscoring the need for continued innovation in strategies for soft tissue reconstruction and rehabilitation. Future studies should focus on identifying predictors of instability and developing standardized protocols for its prevention and management, while maintaining attention to long-term function and patient-reported outcomes. Ultimately, reducing the risk of postoperative instability is key to improving the overall success and durability of proximal humerus reconstruction in oncologic patients.

## Figures and Tables

**Table 3:** Scoring system.

Scoring System	No. of Studies	Reported Range
MSTS (Musculoskeletal Tumor Society score)	12	12/30 (40%)–27/30 (90%)
DASH (Disabilities of the Arm, Shoulder, and Hand score)	3	20.8 (range 2.5–35.8) to 61.4 in unstable cases
DASH (stable vs. unstable shoulders)	2 (36 pts)	Stable: 26.6; Unstable: 61.4
MSTS (stable vs. unstable shoulders)	2	Stable: 75.8%; Unstable: 45.6%
Other scores (Constant, ASES, Enneking, ISOLS)	<2 each	–

## Data Availability

The original contributions presented in this study are included in the article/Supplementary Material. Further inquiries can be directed to the corresponding author.

## Supplementary Materials

The following supporting information can be downloaded at: https://www.mdpi.com/article/10.3390/jcm14217850/s1, PRISMA Checklist [39].
